# Diagnostic Utility of ACTH, Cortisol, DHEAS, and Their Derived Ratios in Cushing’s Syndrome Subtypes

**DOI:** 10.3390/jcm15072772

**Published:** 2026-04-07

**Authors:** Ekin Yiğit Köroğlu, Abbas Ali Tam, Sevgül Faki, Pervin Demir, Fatma Neslihan Çuhaci Seyrek, Didem Özdemir, Oya Topaloğlu, Reyhan Ersoy, Bekir Çakir

**Affiliations:** 1 Endocrinology and Metabolism Diseases Department, Ankara Bilkent City Hospital, 06800 Ankara, Turkey; 2 Endocrinology and Metabolism Diseases Department, Faculty of Medicine, Ankara Yıldırım Beyazıt University, 06800 Ankara, Turkey; 3 Bioistatistics and Medical Informatics Department, Faculty of Medicine, Ankara Yıldırım Beyazıt University, 06800 Ankara, Turkey

**Keywords:** Cushing’s syndrome, adrenocorticotropic hormone, dehydroepiandrosterone sulfate, cortisol, Cushing’s disease, mild autonomous cortisol secretion

## Abstract

**Background/Objectives**: Differentiating Cushing’s disease (CD) from adrenocorticotropic hormone (ACTH)-independent Cushing’s syndrome (AICS) remains challenging in patients with equivocal ACTH levels. While dynamic testing is frequently required, baseline hormonal measurements may offer a simpler diagnostic approach. We aim to evaluate the diagnostic value of baseline plasma ACTH, cortisol, and dehydroepiandrosterone sulfate (DHEAS) levels and their derived ratios for differentiation between ACTH-dependent and ACTH-independent Cushing’s syndrome, and to propose a diagnostic algorithm based on these parameters. **Methods**: This retrospective single-centre study included adult patients with endogenous Cushing’s syndrome aged 18–75 years who were followed at our institution. Patients with ectopic/paraneoplastic Cushing’s syndrome were excluded. The AICS group comprised overt adrenal CS and mild autonomous cortisol secretion cases. Morning baseline plasma ACTH (pg/mL), serum cortisol (µg/dL), and serum DHEAS (µg/dL) levels were measured and ratios calculated: cortisol-to-ACTH ratio (CAR), DHEAS-to-cortisol ratio (DCR), and CAR-to-DHEAS ratio (CAR/D). ROC analysis assessed diagnostic performance with age and sex adjustments. **Results**: A total of 100 patients were included, comprising 43 patients with CD and 57 with AICS. Plasma ACTH demonstrated high diagnostic accuracy for identifying CD with a cut-off of ≥14.65 pg/mL (sensitivity 100%, specificity 98.25%, AUC 0.998). Serum DHEAS showed strong discriminative power with a cut-off of ≥67.15 µg/dL (sensitivity 88.37%, specificity 91.23%, AUC 0.925), achieving high discriminative power after age–sex adjustment at ≥85.59 µg/dL (sensitivity 100%, specificity 100%, AUC 0.999). CAR showed good performance in identifying CD with a cut-off of ≤0.75 µg/dL per pg/mL (sensitivity 93.02%, specificity 98.25%, AUC 0.980). CAR/D demonstrated high diagnostic power with a cut-off of ≤1.54 (sensitivity 95.35%, specificity 98.25%, AUC 0.974), improving after age–sex adjustment to ≤2.36 (sensitivity 97.87%, specificity 96.23%, AUC 0.992). **Conclusions**: Baseline plasma ACTH, serum cortisol, and serum DHEAS measurements, along with derived ratios—especially CAR and CAR/D—provide highly accurate differentiation between ACTH-dependent and ACTH-independent Cushing’s syndrome. These widely available measurements may reduce dependence on dynamic testing and improve diagnostic accuracy in patients with equivocal findings.

## 1. Introduction

Endogenous Cushing’s syndrome (CS) etiology is evaluated under two groups: Adrenocorticotropic hormone (ACTH)-dependent CS (ADCS) and ACTH-independent CS (AICS). ADCS results from hypercortisolemia originating from the pituitary gland or an ectopic focus such as a tumour. AICS is a condition that originates from the adrenal gland [1]. A differential diagnosis should be made in order to identify the source of cortisol excess and direct appropriate treatment options [2].

In the differential diagnosis of endogenous CS, the first test to be performed after screening tests confirm the diagnosis is ACTH measurement [3]. A low or suppressed ACTH measurement of <10 pg/mL suggests AICS, whereas > 20 pg/mL favours ADCS. In cases where measurement results between 10 and 20 pg/mL are obtained and considered equivocal, dynamic tests and imaging methods are utilized [1,2]. This equivocal range can also be accepted as ACTH measurements between 5 and 20 pg/mL in the literature [4,5]. Thirty percent of patients with CS are seen in this broad equivocal range. This leads to the need for repeated ACTH measurements, advanced imaging methods, and dynamic tests [6].

The prevalence of adrenal incidentaloma in autopsy series ranges from 1% to 8.7% [7]. A total of 40–70% of these masses present as non-functioning, while 20–50% are associated with mild autonomous cortisol secretion and 1–4% with overt Cushing’s syndrome [8]. In addition, although pituitary magnetic resonance imaging (MRI) is the most sensitive imaging modality for the detection of a pituitary adenoma, it may still occasionally fail to localize an existing lesion. Depending on the centre where the imaging is carried out, the identification of a sellar image compatible with the diagnosis of corticotropinoma varies from 40% to 90% [9]. Therefore, radiological findings should be interpreted together with biochemical and clinical data during the differential diagnosis process.

Patients’ baseline hormone levels can be assessed more effectively to reduce the potential for confounding by imaging modalities and the need for dynamic testing, which may require hospitalization and additional diagnostic costs. Various studies have been carried out in this direction. A study including 53 CS patients showed that a differential diagnosis could be made with a sensitivity of 100% and a specificity of 86.7% with a threshold value of 12.6 pg/mL for ACTH [10]. Measurement of dehydroepiandrosterone sulphate (DHEAS) has also been considered helpful in determining the cause of CS. A multicenter study of 623 CS patients showed that a DHEAS cut-off value of less than 20% of the reference interval could be used for the differential diagnosis of CD and adrenal CS with high sensitivity and specificity [11].

Our aim in this study is to determine reliable cut-offs and to establish more effective evaluation methods in using ACTH, cortisol and DHEAS values required in the initial evaluation to elucidate the etiology of hypercortisolemic patients.

## 2. Materials and Methods

### 2.1. Study Design

This was a retrospective single-centre study conducted at the Endocrinology Department of Ankara Bilkent City Hospital between December 2019 and January 2025.

### 2.2. Study Population

Patients who were followed-up with a diagnosis of endogenous CS were included in the study, including those with pathologically confirmed Cushing’s disease and those with AICS. The AICS group included patients with overt adrenal CS and mild autonomous cortisol secretion (MACS). Patients with adrenal lesions without overt signs and symptoms of CS and with a serum cortisol concentration >1.8 µg/dL after suppression with dexamethasone (overnight 1 mg and/or 2 days 2 mg) were considered MACS [8]. Patients under 18 years of age and patients with ectopic CS were excluded. Patients using medications that could interfere with dexamethasone metabolism or cortisol assay interpretation were also excluded from the study. In addition, patients who were followed-up for endogenous CS but were not sampled for ACTH, cortisol and DHEAS at the appropriate time and under the proper conditions were excluded from the study, especially those with prolonged sample transfer for ACTH.

### 2.3. Study Protocol

The study included patients’ demographic and clinical data, including age, sex and results of dexamethasone suppression tests (DST) (1 mg overnight DST and 2 days low-dose DST) for CS. Due to differences in assays used at different times between patients, 24 h urinary cortisol and nocturnal salivary cortisol measurements were not included in the study. Basal ACTH (pg/mL), cortisol (µg/dL) and DHEAS (µg/dL) levels were collected. All these measurements were taken at 08.00 a.m. in the fasting state under appropriate conditions. As part of our centre’s standardized evaluation protocol for Cushing’s syndrome, all patients undergo comprehensive baseline hormonal assessment including ACTH, cortisol, and DHEAS measurements at initial presentation, regardless of suspected etiology. These parameters were measured simultaneously for each patient. The cortisol/ACTH formula calculated the ratio of cortisol to ACTH (CAR), the ratio of DHEAS to cortisol (DCR) by the DHEA-S/Cortisol formula, and the ratio of CAR to DHEAS (CAR/D) by the (CAR/DHEAS) × 100 formula.

Serum cortisol levels were analyzed by chemiluminescence immunoassay with a Simens cortisol kit (Simens Atellica HormoneAnalyzer, Tarrytown, NY, USA), and intra- and inter-assay coefficients of variability (CVs) were 3.0% and 4.7%, respectively. The morning serum cortisol level reference range was 5.2–22.4 µg/dL. For ACTH measurements, plasma samples were kept in frosted silicon glass tubes containing ethylenediaminetetraacetic acid (EDTA) and stored below −20 °C until transfer. The morning plasma ACTH level reference range was 7–46 pg/mL. Serum DHEAS levels were analyzed by chemiluminescence immunoassay (Simens Atellica HormoneAnalyzer, Tarrytown, NY, USA), and inter-assay and intra-assay CVs < 10%. The reference range for serum morning DHEAS was 34.5–568.9 µg/dL (manufacturer’s reference for the primary age group, age- and sex-specific adjustments were incorporated in statistical analyses).

### 2.4. Statistical Analysis

Assuming a two-sided test with a 95% confidence level and 90% power, the required minimum sample size was determined to be 40 participants per group (CD and AICS) to achieve 90% power for detecting an area under the curve (AUC) of 0.70 [12]. The threshold of 0.70 was selected based on the findings of Iwamoto et al. [13], which identified it as a critical value for distinguishing between CS subtypes.

ACTH values below the assay’s limit of detection (LOD = 5 pg/mL) were observed in 12 patients (12% of cohort; all in AICS group: 9 adrenal CS, 3 MACS). These were substituted using the LOD/√2 approach (3.54 pg/mL), a recommended method for data with low censoring rates [14]. Sensitivity analysis using alternative imputations (LOD value: 5 pg/mL) yielded similar ROC results confirming robustness of findings. Calculations and statistical analyses were performed using the R programming language (R core team, 2024, version 4.3.2), with the “pROC” package [12], while graphs were created using the “ggplot2” [15] and “magick” packages [16]. A significance level of *p* < 0.05 was considered statistically significant. Descriptive statistics were presented as mean ± standard deviation, median (quartile 1–quartile 3) for continuous variables, and frequency (percentage) for categorical variables. The distribution of variables was assessed using the Shapiro–Wilk test to evaluate normality. Group comparisons were performed using the Pearson Chi-square test for gender and the Kruskal–Wallis test for other variables based on data distribution. Post hoc pairwise comparisons were performed with Bonferroni correction to adjust for multiple comparisons and control the family-wise error rate. The diagnostic accuracy of the ACTH, Cortisol, CAR, DHEAS, CAR/DHEAS and DCR in distinguishing CD from AICS was evaluated using receiver operating characteristic (ROC) curves and the area under the curve (AUC) to assess performance. To account for potential confounding by age and sex, we performed adjusted ROC analysis using logistic regression. For each biomarker, a multivariable logistic regression model was constructed. Adjusted ROC curves were generated using predicted probabilities from these models. The AUC from adjusted curves represents the discriminative ability after controlling for age and sex differences between groups. Optimal cut-off values for both standard and adjusted ROC curves were determined using Youden’s index. DeLong’s test assessed whether age–sex adjustment significantly improved discriminative performance.

## 3. Results

### 3.1. Baseline Data of the Study Groups

A total of 100 patients were included in the study, including 43 patients with CD and 57 patients with AICS. The study sample comprised 43% individuals with CD and 57% with AICS. Among the AICS group, 53% were classified as Adrenal CS and 47% as MACS. In the CD group, 90.7% of participants were female, compared to 78.9% in the AICS group (Table 1). Gender distribution was similar among the CD, Adrenal CS, and MACS groups (*p* = 0.277). Age was similar between the CD and adrenal CS groups (*p* > 0.05) but was statistically significantly higher in the MACS group than in both other groups (*p* = 0.001 vs. CD; *p* = 0.024 vs. adrenal CS).

After 1 mg overnight DST, cortisol levels were statistically significantly higher in the CD group than in the AICS group (14.97 ± 11.16 µg/dL vs. 7.24 ± 7.58 µg/dL, *p* < 0.001). Similarly, cortisol levels after 2 days of low-dose DST were statistically significantly higher in the CD group (12.51 ± 8.25 vs. 7.37 µg/dL ± 7.01 µg/dL, *p* < 0.001).

ACTH, cortisol, CAR, DHEAS, CAR/D, and DCR levels were statistically significantly different between the CD and adrenal CS groups (all *p* < 0.001). These parameters were also statistically significantly different between the CD and MACS groups (all *p* < 0.001). In contrast, ACTH, cortisol, CAR, DHEAS, CAR/D, and DCR levels were similar between the adrenal CS and MACS groups (all *p* > 0.05).

A total of 49 patients (49%) had ACTH < 10 pg/mL (all AICS: 13 MACS and 36 adrenal CS), 12 patients (12%) had ACTH 10–20 pg/mL (equivocal results: 4 CD, 4 MACS, 4 adrenal CS), and 39 patients (39%) had ACTH > 20 pg/mL (all CD).

### 3.2. ROC Analysis

**Figure 1:**
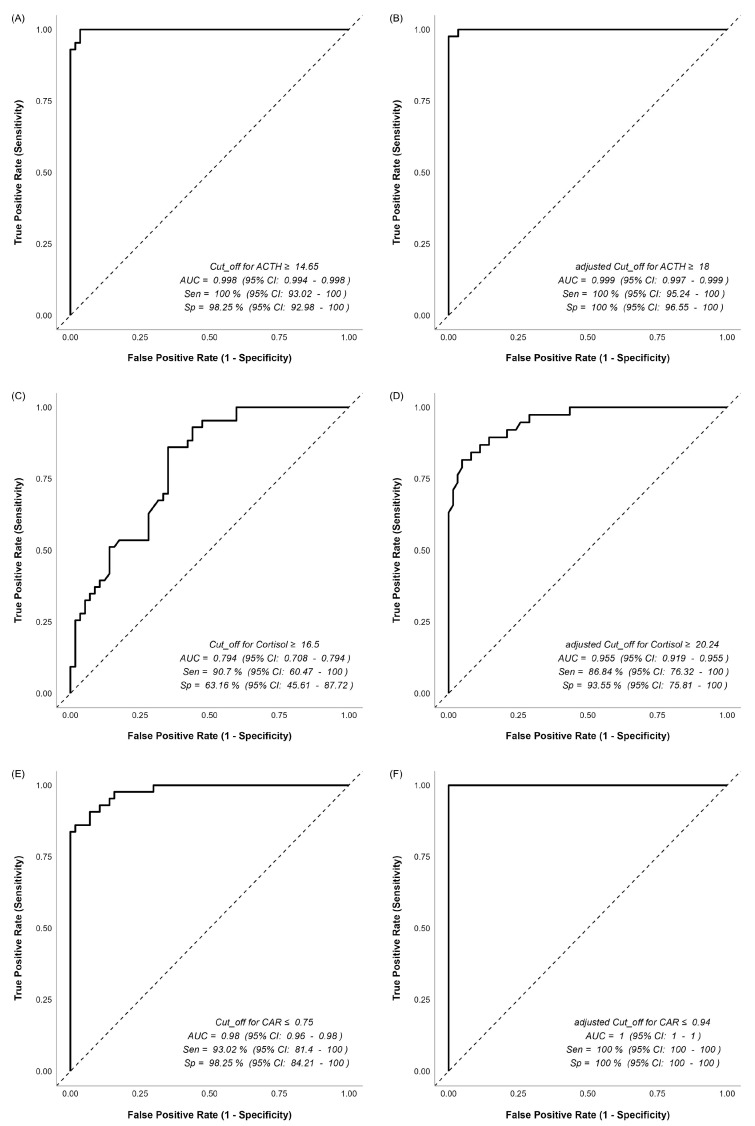
Receiver operating characteristic (ROC) and age–gender-adjusted ROC curves for ACTH, cortisol, and CAR. (**A**) ROC curve for ACTH; (**B**) age–gender-adjusted ROC curve for ACTH; (**C**) ROC curve for cortisol; (**D**) age–gender-adjusted ROC curve for cortisol; (**E**) ROC curve for CAR; (**F**) age–gender-adjusted ROC curve for CAR.

**Figure 2:**
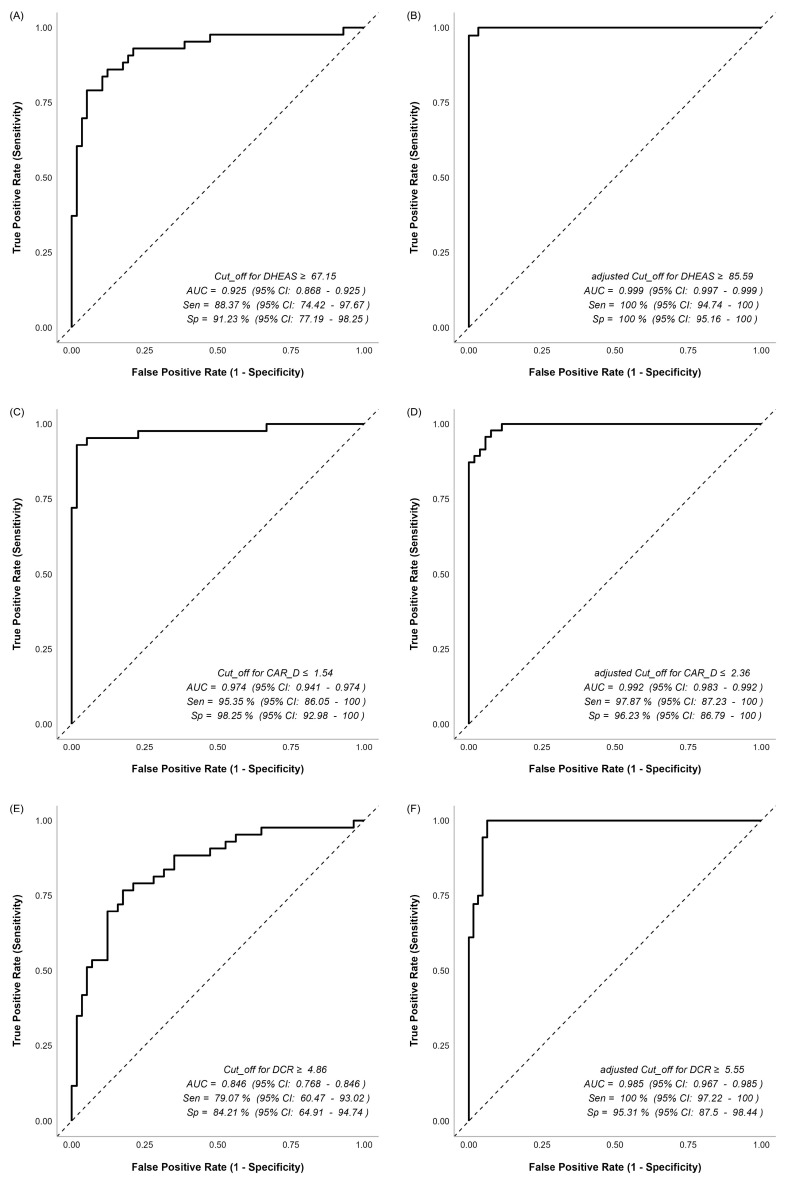
ROC and age–gender-adjusted ROC curves for DHEAS, CAR/D, and DCR. (**A**) ROC curve for DHEAS; (**B**) age–gender-adjusted ROC curve for DHEAS; (**C**) ROC curve for CAR/D; (**D**) age–gender-adjusted ROC curve for CAR/D; (**E**) ROC curve for DCR; (**F**) age–gender-adjusted ROC curve for DCR.

The ROC and adjusted ROC results for all variables are presented in Figure 1 and Figure 2. All AUC values obtained from the ROC analysis are statistically significant (*p* < 0.05). No significant differences were observed between the ROC and adjusted ROC curves for ACTH (*p* = 0.596), CAR (*p* = 0.055), and CAR/D (*p* = 0.309). However, statistically significant differences were noted for Cortisol (*p* = 0.001), DHEAS (*p* = 0.012), and DCR (*p* = 0.001), indicating that the adjusted ROC curves differ statistically significantly from the standard ROC curves for these variables.

The CAR demonstrated very high discriminative performance within this cohort, with an AUC of 0.980 (95% CI: 0.960–0.980) and a cut-off of ≤0.75, showing high sensitivity (93.02%) and specificity (98.25%) for distinguishing between CD and AICS. After adjusting for age and gender, the diagnostic performance remained similar, with an AUC of 1.000 and 100% sensitivity and specificity, although the difference was not statistically significant (*p* = 0.055). Similarly, the CAR/D exhibited strong discriminative power, with an AUC of 0.974 (95% CI: 0.941–0.974) and a cut-off of ≤1.54, demonstrating high sensitivity (95.35%) and specificity (98.25%) for diagnosis of CD. After adjustment for age and gender, the AUC slightly improved to 0.992 (95% CI: 0.983–0.992), with a sensitivity of 97.87% and specificity of 96.23%, but the difference was not statistically significant (*p* = 0.309). The DCR also showed substantial diagnostic accuracy, with an AUC of 0.846 (95% CI: 0.768–0.846) and a cut-off of ≥4.86, sensitivity of 79.07% and specificity of 84.21%. After adjustment, the AUC increased significantly to 0.985 (95% CI: 0.967–0.985), with 100% sensitivity and 95.31% specificity, demonstrating a statistically significant improvement (*p* = 0.001).

Among the 100 patients, 12 (12%) presented with ACTH values in the traditional grey zone (10–20 pg/mL): four with CD and eight with AICS. Due to the small and unbalanced sample size in this subgroup (n = 4 vs. n = 8), formal statistical comparison was not feasible as it would lack adequate power and reliability. However, descriptive analysis showed that all four CD patients had CAR values ≤ 0.75, while 7/8 (87.5%) AICS patients had CAR values > 0.75, suggesting maintained discriminative capacity of CAR even in this challenging diagnostic scenario. Similarly, all four CD patients had DHEAS levels > 85.59 µg/dL, while all eight AICS patients had DHEAS levels < 85.59 µg/dL, providing further etiological differentiation beyond ACTH measurement alone.

## 4. Discussion

This study shows that ACTH, cortisol and DHEAS can be used effectively with defined cut-offs to determine the cause of endogenous CS. In addition, additional diagnostic parameters were established with the ratios of these measurements to each other. Their use alone or in combination may benefit the clinician as additional tools in managing patients with equivocal ACTH results.

The female predominance and mean age of CD observed in this study are similar to those reported in the literature. The mean age for CD was 42 years in the study by Agustsson et al. [17], 48 years in the study by Wengander et al. [18], and 45 years in this study. For adrenal CS, the mean age was 48 years in this study, while similarly, the mean age was 46 years in a study conducted in Sweden [18]. The mean age of patients enrolled in the study as MACS was higher than in the other groups. In a cohort of 84 patients with MACS, the mean age of the patients was 57 years, similar to that in this study [19]. The older age of the patients in the MACS group may be because they were diagnosed late because the clinical findings were mild.

The inclusion of MACS patients alongside overt adrenal CS warrants discussion. While these entities differ in clinical severity, both represent ACTH-independent cortisol secretion with consequent ACTH and DHEAS suppression. Our subgroup analysis shows no significant differences in baseline ACTH, DHEAS, or derived ratios between MACS and adrenal CS patients (Table 1); however, this finding should be interpreted in the context of the limited subgroup sample size. This pragmatic approach reflects real-world practice, where distinguishing autonomous cortisol secretion (regardless of severity) from pituitary-driven hypercortisolism is the initial diagnostic challenge. Therefore, patients with mild MACS were analyzed together with overt adrenal CS. Nevertheless, inclusion of MACS may influence discrimination metrics and contribute to high AUC values. Excluding MACS would substantially reduce sample size and statistical power for ROC analyses, leading to less stable AUC and cut-off estimates. Accordingly, results should be interpreted cautiously, and external validation in independent cohorts is required.

The first step in determining the cause of endogenous CS is the measurement of ACTH levels. However, there is a broad grey zone when assessing ACTH levels [1]. Many studies have been conducted to facilitate patient management by narrowing this area. In our study, a cut-off value of 14.65 pg/mL for ACTH and 18 pg/mL for ACTH, adjusted for age and sex, showed a high diagnostic accuracy. Similarly, Hong et al. [20] showed that a cut-off value of 5 pmol/L (22.7 pg/mL) for ACTH can be used in a study of 92 patients. Another study of 87 CS patients found that ACTH levels < 10 pg/mL may indicate AICS and >30 pg/mL for ADCS. Although this publication is a valuable study showing that a low ACTH threshold of <5 pg/mL is not required for diagnosis of AICS, the number of AICS patients is relatively lower than ADCS patients (n = 15 vs. 72) [21]. In our study, the number of patients is more comparable. In addition, unlike our study, they also included patients with ectopic CS. We did not include ectopic CS patients who were followed-up in our clinic because they had very high ACTH levels. We were concerned that this might be a confounding factor. Still, in that study, it was reported that the ACTH levels of ectopic CS patients were similar to other patients. Because of these differences, the results obtained between studies may differ. A study published in 2024 that included 51 CS patients shows that a cut-off value of 12.6 pg/mL for ACTH could be used in the etiological differentiation of endogenous CS [10]. In this study, similarly to ours, ectopic CS patients were not included. When evaluating the ACTH cut-off values obtained in all these studies, it should be kept in mind that the assays used in each centre may differ, affecting the evaluation.

Another parameter that can be used for the diagnosis and differential diagnosis of endogenous CS is DHEAS [11,20,22,23]. Serum DHEAS levels are expected to be in the normal to high range in CD patients, whereas they are expected to be low in AICS patients due to ACTH suppression [24]. However, studies using DHEAS to make this distinction have produced conflicting results. Hong et al. [20] reported that serum DHEAS levels may overlap in both patient groups and have a lower diagnostic value than serum ACTH. A study of 100 patients published in 2020 showed that a cut-off value of 75.9 µg/dL (2.06 μmol/L) for DHEAS can be used with 95% sensitivity and 100% specificity for aetiological distinction [22]. In our study, a cut-off value of 85.59 µg/dL, close to this result, was found to have a high diagnostic accuracy. DHEAS levels are known to be influenced by age and sex [25]. In this study, the fact that the cut-offs were determined using age- and sex-adjusted analyses increases the reliability of this result.

Regarding the clinical application of adjusted versus unadjusted cut-offs, the unadjusted thresholds represent our primary analysis and are intended for routine clinical use due to their simplicity and adequate diagnostic performance. Age- and sex-adjusted analyses were performed primarily to explore whether demographic adjustment—particularly relevant for DHEAS, which is strongly influenced by age and sex—would meaningfully enhance discriminative accuracy at a population level. Although adjustment improved the performance of DHEAS-based parameters, this should be interpreted as a methodological refinement rather than as a separate clinical threshold. Therefore, we recommend the use of unadjusted cut-offs in routine practice, while age- and sex-adjusted results should be viewed as supportive information rather than mandatory decision thresholds.

A study by Carafone et al. [26] with 256 patients with autonomous cortisol secretion of adrenal origin shows that DHEAS and ACTH can be used with high diagnostic accuracy to differentiate patients with non-functioning adrenal lesions. This study compared patients under a pathophysiological negative feedback mechanism towards ACTH with patients with hypothalamic–pituitary–adrenal axis functioning in normal physiology. This type of comparison and these findings also support the high diagnostic accuracy of our results. Because, in our study, patients whose ACTH was suppressed by negative feedback were compared with patients with pathologically increased ACTH production. In fact, due to the pathophysiological differences in the patient groups in which AICS patients were compared, it should be expected that evaluations with DHEAS and ACTH would give even more significant results and higher specificity in our study. In the end, this is precisely how the results have been observed.

Baseline cortisol levels alone are not commonly used to differentiate CD from AICS. Similarly, basal cortisol values are not routinely used to distinguish between adrenal CS and MACS patients. Still, it is known that cortisol values measured after DST are higher in adrenal CS patients than in MACS patients [27]. In this study, basal cortisol levels were not significantly different between MACS and adrenal CS patients. However, between the CD and AICS groups, CD patients had higher cortisol levels. However, differentiation by cortisol levels alone had a lower diagnostic accuracy than ACTH and DHEAS. As far as we have seen in the literature, there is no study on the differential diagnosis of endogenous CS with cortisol levels alone. However, we thought that the relationship between cortisol and other hormones could be a guide for clinicians. Therefore, we included CAR, CAR/D and DCRs in the study.

There have been some previous studies on CAR. These studies evaluated its usefulness in diagnosing MACS or CS in patients with adrenal incidentalomas and reported that it was a reliable parameter in these studies [13,28]. The effectiveness of this ratio in diagnosing hypoadrenalism was also evaluated, and it was found that although it predicted hypoadrenalism, its diagnostic power was limited [29]. Our study is the first to test CAR’s usefulness in finding the source of endogenous CS, showing high sensitivity and specificity. As expected, it was also more valuable than cortisol level alone.

DCR or cortisol/DHEAS ratio are parameters that have been assessed in many diseases except for diseases associated with hypercortisolemia [30,31]. DCR was added to this study with the idea that it may be a more sensitive and specific method than DHEAS or cortisol values alone. Since DHEAS is affected by age and gender, when DCR was adjusted according to these factors, it was not more valuable than DHEAS measurement alone. However, it still had a high diagnostic accuracy. The CAR/D ratio was also included in the study as it was considered that it could discriminate between CD and AICS with greater diagnostic power when DHEAS was added to CAR, based on the knowledge that both ACTH and DHEAS would be lower in AICS. Although in our study, the addition of DHEAS to this measurement did not seem to have any additional contribution because CAR already has very high sensitivity and specificity, the fact that the CAR/D ratio also showed high diagnostic accuracy suggests that it is a usable parameter. Moreover, this ratio was defined and tested for the first time in this study.

**Figure 3:**
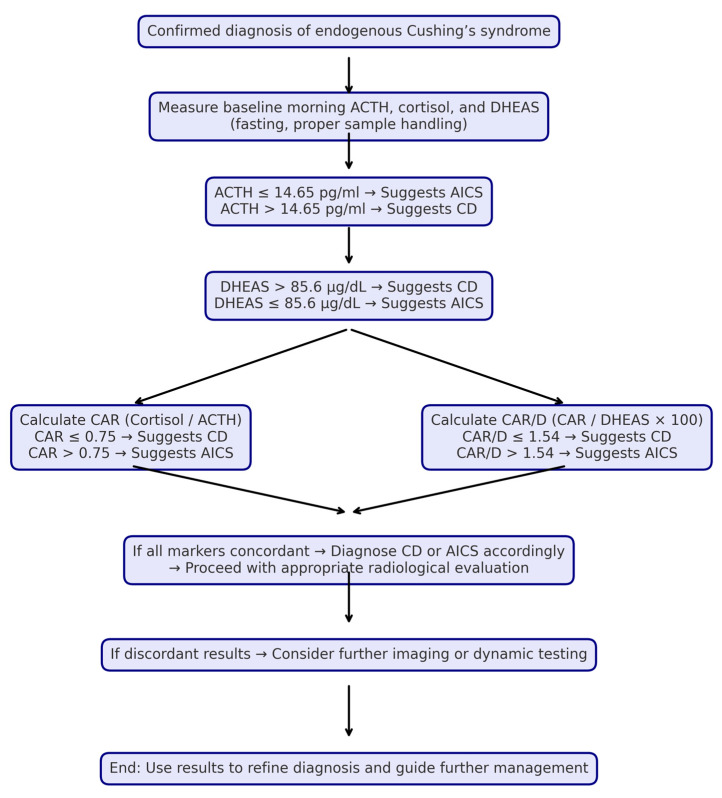
Diagnostic algorithm using ACTH, DHEAS, CAR and CAR/D.

Effective use of these baseline parameters and these ratios in determining the cause of endogenous CS may also support the more accurate interpretation of radiological imaging. Although dynamic pituitary MRI is the most helpful examination in imaging pituitary adenomas, studies have shown that false-positive or false-negative results may be obtained, especially in patients with microadenomas [32,33]. In addition to pituitary MRI, adrenal CT should also be considered in the imaging-based evaluation of Cushing’s syndrome subtypes. Considering the prevalence of adrenal incidentalomas [7], lesions detected on adrenal imaging may not always direct the clinician to the correct source. The fact that imaging methods cannot provide definitive guidance and that a wide grey zone is determined for ACTH and other hormone parameters are not included in the differential diagnosis process also necessitates the performance of additional dynamic tests such as the desmopressin stimulation test or corticotropin releasing-hormone (CRH) test [3]. CRH production is currently suspended. Conflicting results have been obtained regarding the determination of the current diagnostic criteria and the diagnostic power of the desmopressin-based tests that have started to be used instead [34,35,36,37]. Therefore, even including only ACTH, cortisol and DHEAS measurement results and the ratios specified in this study in the diagnosis process may reduce the need for these tests and provide a more accurate evaluation of imaging results. According to the findings obtained, the diagnostic algorithm we can recommend can be seen in Figure 3.

A significant limitation is the relatively small number of patients with ACTH values in the traditional grey zone (10–20 pg/mL; n = 12, 12% of cohort), which precluded adequately powered subgroup analysis. The unbalanced distribution within this subgroup further limited statistical comparisons. While our descriptive findings in these cases were encouraging, prospective studies specifically targeting grey-zone patients are needed to definitively establish the diagnostic utility of hormonal ratios in this clinically challenging population. This represents the primary limitation of our study given that grey-zone cases constitute the most diagnostically challenging scenario where novel biomarkers would have greatest clinical impact.

The study’s limitations include the fact that it was a retrospective study. In addition, if we had the clinical findings of the patients at the time of examination, we could have evaluated the relationship of these hormonal values not only with the etiology but also with the clinical findings. Also, the study was conducted at a single tertiary centre, which may limit generalizability to other populations or practice settings. A significant limitation is the absence of 24 h urinary-free cortisol (24 h-UFC) and late-night salivary cortisol (LNSC) data. Different assays were employed over the study period (2019–2025), precluding standardized comparison. However, we emphasize that our study addresses etiological differentiation of AICS and CD after CS diagnosis has been established, not initial screening. ACTH, cortisol, and DHEAS measurements—when performed under standardized conditions as in our protocol—remain valid for etiological assessment regardless of the screening modality used for initial diagnosis. Future prospective studies incorporating standardized 24 h-UFC and LNSC measurements would strengthen these findings.

The inclusion of MACS patients together with overt adrenal CS in the AICS group represents an important limitation of the present study. Although both conditions belong to the spectrum of ACTH-independent cortisol secretion and are characterized by suppression of ACTH-driven adrenal androgen production, they differ in clinical severity and biochemical intensity. Therefore, combining these entities may have introduced heterogeneity and may have influenced the observed discriminatory performance of ACTH, DHEAS, and derived ratios. In our cohort, subgroup analyses did not show significant differences between MACS and overt adrenal CS for basal ACTH, DHEAS, or the derived ratios; however, these comparisons were limited by subgroup sample size. Accordingly, our results should be interpreted with caution, and future studies should validate these biomarkers in larger cohorts with separate analyses for overt adrenal CS and MACS.

Another important consideration relates to the inclusion of cortisol and cortisol-derived ratios in etiological differentiation. Basal cortisol levels and post-dexamethasone cortisol concentrations are known to reflect disease severity and cortisol burden rather than etiology alone. Therefore, incorporation of cortisol into derived indices such as CAR and DCR may partially capture severity-related differences between groups, potentially contributing to their discriminative performance. This limitation should be acknowledged when interpreting cortisol-based ratios, and these indices should be viewed as adjunctive tools rather than standalone determinants of etiology.

The very high AUC values observed for CAR and CAR/D should be interpreted cautiously. Such levels of discrimination are uncommon for biological markers and may reflect characteristics of this single-centre retrospective cohort, including exclusion of ectopic ACTH syndrome, reliance on ACTH-based classification, and limited sample heterogeneity. These factors may contribute to overestimation of diagnostic performance. Therefore, the present findings should be considered hypothesis-generating and require external validation in independent and more heterogeneous populations.

One of the most critical strengths of the study is that, although it was a retrospective study, it was ensured that hormonal tests were taken at the appropriate times and conditions and studied under appropriate transfer conditions. Undoubtedly, the most crucial point in evaluating these hormonal values is the reliability of the tests. Another strength of the study is that a sufficient number of patients were included for a rare disease such as CS. In addition, some parameters not mentioned in the literature or not used in CS were described or used for the first time in this study.

## 5. Conclusions

In this cohort, basal ACTH showed the strongest diagnostic performance for differentiating CD from AICS. DHEAS and the derived ratios, particularly CAR and CAR/D, provided additional discriminatory value and may support etiological assessment, especially in patients with equivocal ACTH results. These readily available biochemical parameters may also help guide the interpretation of radiological findings and support a practical diagnostic algorithm. However, given the retrospective single-centre design and the inclusion of MACS within the AICS group, these findings should be interpreted cautiously and be externally validated before routine clinical implementation.

## Figures and Tables

**Table 1:** Comparison of values among participants.

Variable	All Subjects (n = 100)	CD (n = 43)	AICS (n = 57)	AICS (n = 57)	
				Adrenal CS (n = 30)	MACS (n = 27)
Female/male	84.0/16.0	90.7/9.3	78.9/21.1	80.0/20.0	77.8/22.2
Age (years)	49.35 ± 13.03 52 (42–58.5)	45.44 ± 14.23 48 (37–53) ^a^	52.3 ± 11.31 54 (45–61.5)	48.2 ± 12.78 49.5 (38.25–57.5) ^a^	56.85 ± 7.23 56 (52–62) ^b^
ACTH (pg/mL)	30.6 ± 36.22 11.15 (5.55–50.43)	62.23 ± 35.75 58.2 (34.1–83.8) ^a^	6.75 ± 3.59 6 (3.54–8.47)	5.82 ± 3.51 3.54 (3.54–6.95) ^b^	7.78 ± 3.46 6.7 (5.7–9) ^b^
Cortisol (µg/dL)	18.8 ± 7.93 17.4 (12.63–22.5)	23.17 ± 7.97 22.1 (17–28.4) ^a^	15.49 ± 6.13 13.3 (10.7–20.24)	15.91 ± 6.57 14.34 (10.7–20.59) ^b^	15.03 ± 5.7 12.7 (10–20) ^b^
CAR	1.81 ± 1.64 1.38 (0.5–2.65)	0.51 ± 0.36 0.47 (0.25–0.58) ^a^	2.8 ± 1.55 2.5 (1.58–3.4)	3.42 ± 1.81 3.27 (2.18–4.56) ^b^	2.11 ± 0.76 2.05 (1.48–2.62) ^b^
DHEAS (µg/dL)	118.13 ± 143.93 59.83 (25.48–175.53)	222.35 ± 166.32 180.72 (91.2–298.19) ^a^	39.51 ± 35.24 29.55 (14.25–54.39)	36.01 ± 42.36 24.33 (13.73–40.72) ^b^	43.39 ± 25.39 41.51 (26.01–60.75) ^b^
CAR/D	8.81 ± 15.29 2.47 (0.29–10.27)	0.76 ± 2.31 0.24 (0.11–0.46) ^a^	14.88 ± 17.95 7.91 (3.56–20.88)	20.33 ± 22.05 10.97 (6.13–27.94) ^b^	8.83 ± 8.92 5.01 (2.41–9.3) ^b^
DCR	6.17 ± 7.03 4.09 (1.54–7.25)	10.25 ± 8.49 7.36 (4.88–12.22) ^a^	3.09 ± 3.28 2.15 (0.8–4.29)	2.9 ± 3.95 1.51 (0.75–3.82) ^b^	3.3 ± 2.37 2.96 (1.26–4.46) ^b^

ACTH: Adrenocorticotropic hormone, CAR: the Cortisol/ACTH ratio, CAR/D: the CAR/DHEAS ratio, DCR: the DHEAS/Cortisol ratio, CD: ACTH-dependent Cushing’s syndrome (Cushing’s disease), AICS: ACTH-independent Cushing’s syndrome, CS: Cushing’s syndrome, MACS: mild autonomous cortisol secretion. Data presented as mean ± standard deviation, median (quartile 1–quartile 3) for quantitative variables, column percentage for gender. The Pearson Chi-square *p*-value for gender was 0.277. The Kruskal–Wallis test results indicate that *p*-values are <0.001 for all other variables. Group differences were assessed using Dunn’s Bonferroni post hoc test. Groups with different letters (a, b) show significant differences (*p* < 0.05).

## Data Availability

The data that support the findings of this study are available on request from the corresponding author.

## Abbreviations

The following abbreviations are used in this manuscript:

ACTH	Adrenocorticotropic hormone
ADCS	ACTH-dependent Cushing’s syndrome
AICS	ACTH-independent Cushing’s syndrome
AUC	Area under the curve
CAR	Cortisol-to-ACTH ratio
CAR/D	CAR-to-DHEAS ratio
CD	Cushing’s disease
CI	Confidence interval
CS	Cushing’s syndrome
CV	Coefficient of variability
DCR	DHEAS-to-cortisol ratio
DHEAS	Dehydroepiandrosterone sulfate
DST	Dexamethasone suppression test
EDTA	Ethylenediaminetetraacetic acid
LOD	Limit of detection
MACS	Mild autonomous cortisol secretion
MRI	Magnetic resonance imaging
ROC	Receiver operating characteristic
